# VOSviewer and Bibliometrix

**DOI:** 10.5195/jmla.2022.1434

**Published:** 2022-07-01

**Authors:** Humberto Arruda, Edison Renato Silva, Marcus Lessa, Domício Proença, Roberto Bartholo

**Affiliations:** 1 humberto.arruda@poli.ufrj.br, Production Engineering Program, Universidade Federal do Rio de Janeiro, Rio de Janeiro, Brazil.; 2 edison@poli.ufrj.br, Industrial Engineering Department and Production Engineering Program, Universidade Federal do Rio de Janeiro, Rio de Janeiro, Brazil.; 3 marcus.lessa@poli.ufrj.br, Production Engineering Program, Universidade Federal do Rio de Janeiro, Rio de Janeiro, Brazil.; 4 domicio.proenca.jr@gmail.com, Production Engineering Program, Universidade Federal do Rio de Janeiro, Rio de Janeiro, Brazil.; 5 bartholo.roberto@gmail.com, Production Engineering Program, Universidade Federal do Rio de Janeiro, Rio de Janeiro, Brazil.

## Abstract

**VOSviewer (version 1.6.17, July 22, 2021).** Centre for Science and Technology Studies, Leiden University, The Netherlands. https://www.vosviewer.com; free, donations accepted.

**Bibliometrix (version 3.1, Sep 24, 2021).** Department of Economics and Statistics, University of Naples Federico II, Italy. info@bibliometrix.org; https://www.bibliometrix.org/; free, donations accepted.

## INTRODUCTION

As the materials on a given topic increases, researchers and librarians find it more difficult to grasp the big picture of a field. Outlining the state of the art, the relationships, opportunities and main players of a given community of practitioners and scholars calls for a map that connects research information, venues, themes, as well as relationships among authors and institutions. The understanding that may emerge from such a big picture is necessary to formulate research, publication, institutional or career strategies. Authors such as Beerepoot et al [1] and Waaijer et al [2] argue that this need has increased with the reduction of the psychological distance of the world, a phenomenon that accelerated during the Covid-19 pandemic. By furthering remote work, many more connections became possible on a worldwide scale. This has led to a change in expectations, that increasingly takes international collaboration and multi-country data for granted, enlarging the requirements of what is to be considered “doing good research”.

## DOMAIN VISUALIZATION

Domain visualization based on citation analysis represents the relationships between sources in a two-dimensional space. This diagram offers a map of the dynamics of the literature and the paths that connect it. This network comprises nodes and edges and admit to customization in order to support a given analysis. Nodes may represent individual pieces of publication, journals, researchers, institutions or keywords. Edges represent the existence or type of relationship between pairs of nodes [3]. This allows the expression of any of a number of possible big pictures of the state of field. It may serve to enlarge the understanding of an individual researcher about the shape and directions of a given subject, it can support the definition of the breadth and reach of the plan for a Systematic Review and avoid biases during the process of source selection [4]. This article will review two of the most popular and promising domain visualization software packages available: VOSviewer and Bibliometrix.

### VOSviewer

VOSviewer was released in 2010 by Nees Jan van Eck and Ludo Waltman (Leiden University) [5]. VOSviewer is a software tool for creating and exploring maps based on network data. While intended primarily for analyzing academic records, it can be used on any type of network data (social networks, e.g.). VOSviewer explores co-authorship, co-occurrence, citation, bibliographic coupling, and co-citation links in one of three possible representations: network, overlay, or density visualization.

### Bibliometrix

Bibliometrix was launched in 2017 by Dr. Massimo Aria (University of Naples Federico II) and Dr. Corrado Cuccurullo (University of Campania Luigi Vanvitelli) [3]. It is a package that must be used within the R software environment. R is both a programming language and a free environment for statistical computing, supported by the R Core Team and the R Foundation for Statistical Computing. Bibliometrix is a comprehensive mapping analysis tool that supports three phases of the biblio-metric analysis process: (i) data import and conversion to R format; (ii) biblio-metric analysis of a dataset and (iii) the construction of matrices. Matrices are customizable and allow mapping in great resolution, being input data for performing network analysis, multiple correspondence analysis, and many other data reduction techniques including domain visualization.

## MAIN PURPOSE

For the purposes of this article, the main concerns of domain visualization correspond to the representation of the attributes and connections of:

- Authors
- Institutions or nations
- Publications
- Sources
- Relationships
- Keywords

## SOFTWARE COMPARISON

VOSviewer and Bibliometrix can be compared succinctly in terms of their installation and usage requirements and of their functionalities (Table 1).

**Table 1:** Comparison of VOSviewer and Bibliometrix.

Item	VOSviewer	Bibliometrix
Software requirements	Linux, Windows or Ma-cOS	Linux, Windows or Ma-cOS
Install requirements	Download the appropriate version for your operating system	Requires installation of R language IDE and then of the Bibliometrix package
Requires programming knowledge	No	Yes
Ease of use	High	Low
Customizability	Low	High
Import data from (file formats accepted)	SCOPUS (CSV), Clari-vate Analytics Web of Science (Plaintext or tab-delimited), Pub-Med/MedLine (MEDLINE) and Dimensions (CSV)	SCOPUS (BibTeX or CSV), Clarivate Analytics Web of Science (Plaintext, BibTeX or EndNote), Pub-Med/MedLine (MEDLINE), Cochrane Database of Systematic Reviews (Plaintext) and RISmed (RIS).
Load multiple files	Yes	Yes
Data analysis from multiple sources	No	Yes
API support	Microsoft Academic, Cross-ref, Europe PMC, Semantic Scholar, the OpenCitations Corpus (OCC), OpenCitations Index of Crossref open DOI-to-DOI citations (COCI), and Wikidata	Dimensions, NCBI PubMed and Scopus
Exports spreadsheets	Yes	No
Dictionary creation (thesaurus)	Yes	No
Exclusive analysis that only one of the tools deliv ers	Temporal evolution of authors and institutions	-Impact indexes (H-in-dex, G-index, M-index) Totalization of authors, sources, base documents
Flexibility and responsiveness to the user	High	Low

VOSviewer is a button-and-window-interface software, confined to its preprogrammed functions and possibilities.

Bibliometrix is a coding terminal that requires knowledge of the R programming language and allows complete customization.

It is important to note that Bibliometrix's developers also created Biblioshiny, a user interface that requires no coding knowledge to simplify usage. However, Biblioshiny does not allow file import of multiple files for the same analysis, imposing the task of merging different files that must be from the same database. This excludes it from consideration in this article.

## FEATURES

### Software requirements

Both Bibliometrix and VOSviewer can be used in the main operating systems (Windows, Linux, and MacOS). VOSviewer was developed in Java, which leads to platform portability. Bibliometrix is a package in the R programming language, which leads to operating system flexibility, but requires an IDE for R, like R Studio.

### Need for programming knowledge

VOSviewer does not require any programming knowledge.

Only Bibliometrix requires the user to have knowledge of programming in R language.

### Ease of use

VOSviewer is immediately accessible with a standard graphical interface.

Bibliometrix requires a customized interface to be programmed, if desired.

### Loading multiple files

The main reference databases limit the export of metadata in a single file. Web of Science allows users to export the metadata of 500 references; Scopus allows 2000. Users have to create more than one file and import them one at a time whenever the number of desired references exceeds the limit.

Both VOSviewer and Bibliometrix allow loading metadata from multiple files.

### Data Analysis from multiple sources

VOSviewer accepts files from different databases, but only one source can be used at a time. Depending on user's requirement, this may be a significant issue, because any one database does not hold the whole literature of any given field: users must combine metadata from different sources.

Only Bibliometrix allows users to analyze data from multiple sources concurrently.

### Flexibility and responsiveness to the user

Flexibility is linked to this diversity of possible manipulation of parameters (colors, lines, labels). Responsiveness to the user is the possibility of seeing changes as they are made without the need to run the program again.

VOSviewer is both more flexible and more responsive than Bibliometrix.

### API support

Both VOSviewer and Bibliometrix allow using an API to automate the communication with the indexing databases (Web of Science, PubMed etc.), keeping the database updated automatically.

## ANALYSIS THAT ONLY ONE OF THE SOLUTIONS DELIVERS

### VOSviewer

#### Spreadsheet export

This functionality allows the data used to create bibliometric maps to be exported to spreadsheets, easing the usual clutter of domain visualization to identify relationships and nodes that might otherwise be missed.

#### Creation of thesaurus

Due to the diversity of keywords in the documents, it is common to have to deal with the occurrence of different words that have the same meaning, as simply as the use of singular and plural in keywords. A thesaurus makes it possible to deal with this problem, allowing references with keywords that are synonymous to be addressed jointly.

#### Temporal Data Visualization

**Figure 1:**
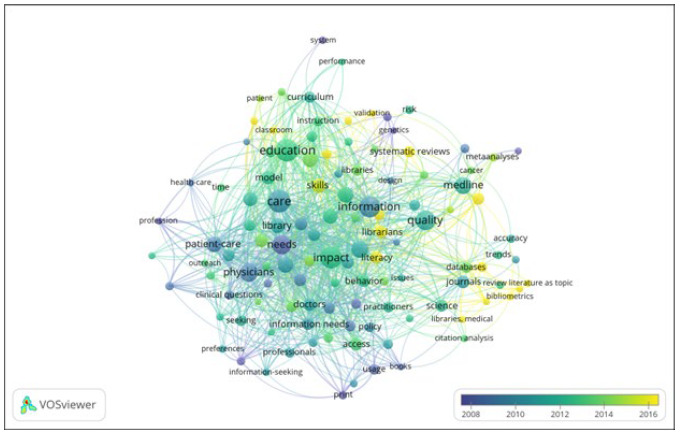
Overlay map of keyword co-occurrence over time for papers published at the Journal of the Medical Library Association (JMLA).

Note: Metadata includes all 1826 articles in the Journal of the Medical Library Association (JMA) (references), retrieved from Web of Science [September 7th, 2021].

The visualization of temporal data can color code network nodes to the year of publication allowing the identification of trajectories and trends in a given field (Figure 1, for keywords, for example).

### Bibliometrix

#### H-index, G-index, M-index

The impact indexes of sources and authors (H-index, G-index, M-index)

#### Production over Time

A map of authors' production over the time.

#### Total Numbers

The total of authors, sources and publications from the database used.

## CONCLUSION

VOSviewer and Bibliometrix are fit for purpose in providing domain visualization, yet each offers a distinctive set of capabilities and requirements.

VOSviewer is the more user-friendly offering simplicity, flexibility and responsiveness to user demands as well as greater graphic quality at the price of bounding alternatives to its preprogrammed functions and requiring repeating analysis due to its inability to combine data from different sources.

Bibliometrix is the more robust and versatile, being capable of greater customization by users and of (a) performing analyses using files from multiple databases, (b) accepting files from the Cochrane Database of Systematic Reviews (CDSR) and from RISmed, (c) offering exclusive analyzes at the price of a steeper learning curve that includes programming.

It can be argued that each fills a particular niche. VOSviewer may be all that a given user or set of users need. However, as a particular capability is required, it may turn out to be a first step before deciding on the extra effort that Bibliometrix will demand.
